# Deep Learning–Assisted Quantification of Iron Oxide Nanoparticles in Magnetic Particle Imaging

**DOI:** 10.51894/001c.164005

**Published:** 2026-07-31

**Authors:** Ossaid Ahmed, Saumya Nigam, Dong Chen, Elvira Gjelaj, Ping Wang

**Affiliations:** 1 College of Osteopathic Medicine, Michigan State University

## 08

### Introduction

Magnetic Particle Imaging (MPI) is an emerging, radiation-free imaging modality that directly detects superparamagnetic iron oxide nanoparticles, enabling high sensitivity and true tracer quantification without tissue background signal. Accurate iron quantification is critical for applications such as cell tracking, drug delivery monitoring, and oncology research. However, current quantification approaches often rely on calibration standards and are susceptible to variability and observer bias. Artificial intelligence (AI), particularly deep learning, offers potential to improve standardization and predictive accuracy in medical image analysis.

### Objectives

We aimed to develop and validate a three-dimensional convolutional neural network (3D CNN) to accurately predict iron content from volumetric MPI data and evaluate its performance in both phantom and in vivo mouse models.

### Methods

A dataset of 175 three-dimensional MPI scans from 3D-printed phantoms containing known quantities of iron (6–800 μg) was used for model training with 10-fold cross-validation. An independent in vivo dataset of 20 CD1 mice injected subcutaneously with known iron quantities served as a testing cohort. Images were preprocessed for intensity normalization and noise suppression. A lightweight 3D CNN regression model was trained to predict iron content. Model performance was assessed using Pearson correlation coefficient (PCC), root mean squared error (RMSE), and mean absolute error (MAE).

### RESULTS

For phantom datasets (≤400 μg), the model achieved a mean PCC of 0.90, RMSE of 48.4 μg, and MAE of 30.6 μg across cross-validation folds. Performance remained strong when including higher iron concentrations, though with increased error variability. In vivo testing demonstrated a PCC of 0.83–0.88 with RMSE of approximately 31.7 μg and MAE of 20.2 μg, indicating consistent predictive accuracy in biological samples.

### Discussion

This study demonstrates that a 3D CNN can reliably quantify iron content from MPI scans, reducing dependence on manual calibration and potentially minimizing observer variability. By advancing automated and standardized quantitative analysis, this approach strengthens the translational potential of MPI and highlights the growing role of AI in improving precision imaging within medical research and future clinical practice.

